# Supplementary material for: Transcriptional Regulation of Amino Acid Transport in Glioblastoma Multiforme
Source: Cancers (Basel). 2021 Dec 7;13(24):6169. doi: 10.3390/cancers13246169 (PMC8699180; doi:10.3390/cancers13246169)
Supplement: Supplementary file 1 [file cancers-13-06169-s001.zip › cancers-1479099-supplementary.pdf]

cancers

MDPI

Transcriptional Regulation of Amino Acid Transport in Glioblastoma Multiforme

Robyn A. Umans [1], Joelle Martin [1], Megan Harrigan [1], Dipan Patel [2], Lata Chaunsali [2], Aarash Roshandel [3], Kavya Iyer [4], Michael D. Powell [5], Ken Oestreich [6] and Harald Sontheimer [2*]

[1] Center for Glial Biology in Health, Disease, and Cancer, The Fralin Biomedical Research Institute at VTC, Roanoke, VA, 24016, USA
[2] Department of Neuroscience, University of Virginia School of Medicine, Charlottesville, VA 22903, USA
[3] College of Agriculture and Life Sciences, Virginia Polytechnic Institute and State University, Blacksburg, VA 24061, USA
[4] Roanoke College, Salem, VA, 24153, USA
[5] Department of Microbiology and Immunity, Emory University School of Medicine, Atlanta, GA, 30322, USA
[6] Microbial Infection and Immunity, The Ohio State University College of Medicine, Columbus, OH 43210, USA
* Correspondence: sontheimer@virginia.edu

Supplementary Materials:

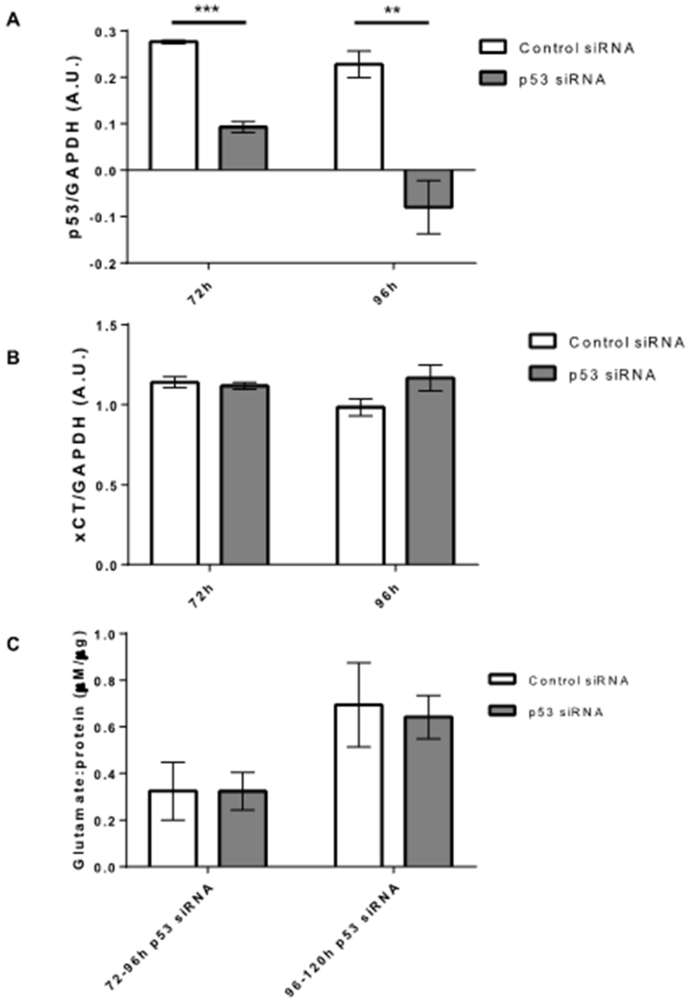

Supplemental Figure S1. p53 knockdown via siRNA does not affect xCT protein levels or glutamate release. Western blot was performed after p53 knockdown with siRNA in GBM14 cells. (**A**) p53

protein is significantly reduced after 72 and 96 hours post-siRNA transfection. (**B**) xCT protein is not significantly reduced after 72 and 96 hours post-siRNA transfection. (**C**) Glutamate release is not significantly reduced after 72 and 96 hours post-siRNA transfection. A two-way ANOVA, with multiple comparison's post hoc Sidak's test was performed. Error bars are graphed with mean + S.E.M. Experiments represent at least three biological replicates. (**B**) *** p =< 0.001, ** p =< 0.01.

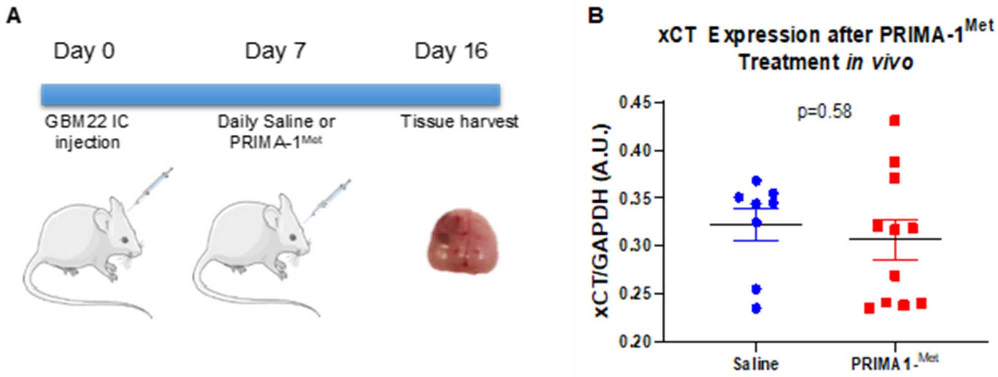

Supplemental Figure S2. PRIMA-1^{Met} does not affect tumor xCT expression in an intracranial tumor model. (**A**) A schematic representing the intracranial tumor implantation and dosing regimen for our PRIMA-1^{Met} *in vivo* experiment. There were 8-11 animals were dosing group. Tumor tissue was harvested after 9 days of treatments. (**B**) xCT protein expression after treatment was determined via Western blots for both groups. There was no change in xCT expression after PRIMA-1^{Met} treatment, which may be attributed to poor blood-brain barrier permeability. An unpaired, two-tailed t-test was performed. Error bars are graphed with mean + S.E.M.
